# A Computerized Test of Design Fluency

**DOI:** 10.1371/journal.pone.0153952

**Published:** 2016-05-03

**Authors:** David L. Woods, John M. Wyma, Timothy J. Herron, E. William Yund

**Affiliations:** 1 Human Cognitive Neurophysiology Laboratory, VANCHCS, 150 Muir Rd., Martinez, CA 95553, United States of America; 2 UC Davis Department of Neurology, 4860 Y St., Suite 3700, Sacramento, CA 95817, United States of America; 3 Center for Neurosciences, UC Davis, 1544 Newton Ct., Davis, CA 95616, United States of America; 4 UC Davis Center for Mind and Brain, 202 Cousteau Place, Suite 201, Davis, CA 95616, United States of America; 5 NeuroBehavioral Systems, Inc., 15 Shattuck Square, Berkeley, CA 94704, United States of America; University of California, San Francisco, UNITED STATES

## Abstract

Tests of design fluency (DF) assess a participant’s ability to generate geometric patterns and are thought to measure executive functions involving the non-dominant frontal lobe. Here, we describe the properties of a rapidly administered computerized design-fluency (C-DF) test that measures response times, and is automatically scored. In Experiment 1, we found that the number of unique patterns produced over 90 s by 180 control participants (ages 18 to 82 years) correlated with age, education, and daily computer-use. Each line in the continuous 4-line patterns required approximately 1.0 s to draw. The rate of pattern production and the incidence of repeated patterns both increased over the 90 s test. Unique pattern z-scores (corrected for age and computer-use) correlated with the results of other neuropsychological tests performed on the same day. Experiment 2 analyzed C-DF test-retest reliability in 55 participants in three test sessions at weekly intervals and found high z-score intraclass correlation coefficients (ICC = 0.79). Z-scores in the first session did not differ significantly from those of Experiment 1, but performance improved significantly over repeated tests. Experiment 3 investigated the performance of Experiment 2 participants when instructed to simulate malingering. Z-scores were significantly reduced and pattern repetitions increased, but there was considerable overlap with the performance of the control population. Experiment 4 examined performance in veteran patients tested more than one year after traumatic brain injury (TBI). Patients with mild TBI performed within the normal range, but patients with severe TBI showed reduced z-scores. The C-DF test reliably measures visuospatial pattern generation ability and reveals performance deficits in patients with severe TBI.

## General Introduction

Jones-Gotman and Milner [1] introduced the design fluency (DF) test to compare the effects of left and right hemisphere lesions in a non-verbal fluency task. Patients spontaneously generated line drawings in the first part of the test, and then drew four-line figures in the second. Patients with cortical lesions produced fewer drawings and more apparent repetitions than control subjects, with the largest decrements seen in patients with right frontal lesions.

Subsequently, Regard et al. [2] developed the five-point test (5-PT) to improve the objectivity of DF testing. In the 5-PT, five dots are arranged in a square frame, with 40 such squares on each response sheet. Participants are instructed to connect two or more dots in each pattern with a pencil, while avoiding repeated patterns. Table 1 summarizes data from recent large-scale 5-PT studies in tests of two [3] to three minute durations [3–7]. Santa Maria et al. [8] quantified the number of patterns produced during each minute of a 10-minute version of the 5-PT and found that the number of patterns produced declined from 15 in the first minute to 5.1 in the tenth minute, with the decline accompanied by an increasing incidence of repetitions. Participants produced an average of 74.2 patterns over 10 minutes, a small fraction of the 1,023 possible patterns (including 10 unique one-line patterns and 45 unique two-line patterns).

**Table 1:** Normative data in design fluency studies.

5-PT	Test	No.	Age	Edu	UPs	SD	CV	Dur	UP/min	RPs	% RPs
Santa Maria et al, 2001	5-pt	80	23.96	15.50	74.22			10	7.42	11.73	13.65%
Fernandez et al., 2009	5-pt	212	47.80	13.10	26.63	9.71	36.46%	3	8.88	2.40	9.00%
Goebel et al., 2009	5-pt	280	44.90	13.30	32.81			3	10.94		8.39%
Cattelani et al, 2011	5-pt	332	37.20	13.11	34.03	8.72	25.62%	3	11.34	2.58	7.59%
Tucha et al, 2012	5-pt	608	41.80	11.80	28.91	9.34	32.32%	2	14.45	1.63	5.34%
Khalil, 2010	5-pt	215	27.40	11.90	32.14	6.84	21.28%	3	10.71	4.38	11.99%
**RFFT**											
Ruff et al. 1978	RFFT	358	44.30	14.40	93.67			5	18.73	9.01	8.77%
Izaks et al, 2011	RFFT	1,651	54.00	13.00	70.00	26	37.14%	5	14.00	7.7	9.91%
Ross, 2014	RFFT	102	21.70	14.00	93.72	21.62	23.07%	5	18.74	5.67	5.70%
van Eersel et al, 2015	RFFT	2515	53.00	14.00	73.00	26	34.93%	5	14.60	7	8.75%
**D-KEFS DF**											
Wecker et al, 2005	D-KEFS	719	50.96	13.30	9.73	3.67	35.14%	1	9.73		
Kramer et al, 2007	D-KEFS	36	64.40	17.20	11.10	3.9	35.14%	1	11.10		
Possin et al, 2012	D-KEFS	37	64.70	16.80	20.10	4.3	21.39%	2	10.05	1.6	7.37%
**Current Experiments**											
Exp. 1	C-DF	180	41.40	14.50	11.54	3.29	28.55%	1.5	7.69	1.61	12.25%
Exp. 2	C-DF	55	26.20	14.80	11.98	2.83	23.62%	1.5	7.99	1.45	10.80%

Abbreviations: 5-pt = five-point test, RFFT = Ruff Figural Fluency Test; D-KEFS = D-KEFS design fluency test; C-DF = computerized design fluency; No. = number of participants; Age = mean age; Edu = years of education; UPs = unique patterns; SD = standard deviation; CV = coefficient of variation; Dur = test duration (min); UP/min = average unique patterns per minute; RP = repeated patterns; % RPs = percentage of total patterns that were repeated.

Ruff et al. [9] developed the Ruff Figural Fluency Test (RFFT), a variation of the 5-PT in which five different asymmetric dot arrangements (some with distractors) are presented on separate response sheets for one minute each. In contrast to the 5-PT, where any repetition over the entire 5-min test is categorized as a perseverative error, unique pattern scores and repetitions are separately tallied for each 1-min test segment in the RFFT. As a result, the average rate of pattern production per minute is higher on the RFFT than the 5-PT [10,11], and repeated patterns occur less frequently (see Table 1). Indeed, young college graduates produced an average of 114 patterns with 7 repetitions during the five-minute test [9,12]. Moreover, in contrast to the 5-PT, the rate of pattern production in the RFFT remains stable across the five test segments [9].

Acceptable patterns on the 5-PT and the RFFT can include drawings with one to four lines that do not need to be connected to one another. In contrast, in the D-KEFS design fluency test [13–16], participants must connect dots using continuous four-line patterns. There are three D-KEFS subtests: a baseline test with five solid circles, a filter test where participants connect open circles and ignore solid circles, and a switching test where participants alternatively connect solid and filled circles. Because each pattern requires four lines, the rate of pattern production in the D-KEFS design fluency test is significantly reduced relative to the rate of pattern production in the 5-PT and RFFT (see Table 1). The rate of pattern production is further reduced in switching conditions.

Intersubject variability is relatively high for all of the design fluency test variants, with coefficients of variation (CVs, SD/mean) ranging from 21.3% [7] to 37.1% [10]. Some of the intersubject variation reflects differences in participant age and education because the rate of pattern production declines with age and increases with education [3,4,6]. As a result, score variance is reduced when normative populations are stratified by age and education [9,10]. The incidence of repeated patterns increases with age in some studies [6,9,10], but not others [3]. Sex does not significantly influence performance on design fluency tests, with the majority of studies finding comparable performance in male and female subjects [4,6,9,10].

Here, we introduce a computerized design fluency (C-DF) test in which subjects connect five dots with continuous four-line patterns using the computer mouse. In Experiment 1, we analyze the demographic factors that influence performance, describe changes in performance over time, and quantify correlations between C-DF test performance and performance on other computerized neuropsychological tests. In Experiment 2, we examine the test-retest reliability of the C-DF test and analyze learning effects across repeated tests. In Experiment 3, we describe the effects of simulated malingering on C-DF performance, and in Experiment 4, we describe C-DF sensitivity in a small group of veteran patients with traumatic brain injury (TBI).

## Experiment 1. Computerized Design Fluency: Normative Data

The results in Table 1 reveal substantial variability in the unique pattern scores of normative populations tested in different laboratories with the same DF test. For example, the mean score of participants in the 5-PT study of Fernandez et al. [5] was nearly one standard deviation below the mean score of participants in the study of Cattelani et al. [4], despite relatively comparable ages (47.8 vs. 37.2 years) and identical levels of education (13.1 years). Similarly, Izaks et al. [10] and van Eersel et al. [11] obtained mean scores with the RFFT that were approximately 0.8 standard deviations below the norms of Ruff et al. [9]. Izaks et al. [10] presented the unique pattern scores stratified into two age ranges and three levels of education from both studies. Statistical analyses of these results showed that each of the six groups in Izaks et al. [10] produced significantly fewer unique patterns than the corresponding group in Ruff et al. [9] [t-values ranged from t(35) = -2.52, p< 0.01 to t(43) = -5.52, p < 0.0001].

The significant differences in unique pattern scores in normative data sets collected in different laboratories may reflect cultural differences, as well as variations in test instruction, administration, and scoring procedures. For example, Lezak [17] suggested that instructions on the RFFT should explicitly emphasize to participants that they produce “patterns” (not “designs”), and stress that participants need to draw only one line to create a pattern. The number of practice trials may also influence performance. In the original RFFT study of Ruff et al. [9], participants were given three practice patterns. However, in more recent studies, examiners have generally omitted practice trials, instead providing several examples of correct patterns [3–6].

Experimenter monitoring of subject performance may also influence outcome. For example, some examiners correct participants after the first pattern repetition [5], while others warn participants not to reproduce the designs that are used for demonstration purposes [4]. Timing measurement may also influence scores. For example, some examiners may start timing after the participant starts drawing the first pattern, whereas others may start timing with the command to “begin”. In addition, examiners may differ as to whether patterns being drawn at test termination are included in the final total.

In manually administered DF tests, examiners must identify patterns and tally the number of unique patterns and pattern repetitions across response sheets. As a result, scoring errors can occur. Although the inter-rater reliability of unique pattern scores is generally high [6,12,18], repeated patterns are less reliably scored, particularly with less experienced examiners [19].

Test administration and scoring are standardized in the C-DF. The C-DF also recorded the time needed to draw each line in the 4-line patterns. Previous studies have shown that the rate of pattern production declines over time on the 5-PT [4,5,8]. We hypothesized that this reduction may reflect a gradual switch from 1- and 2-line patterns to 3- and 4-line patterns in order to avoid pattern repetitions as the test progressed. We therefore predicted more stable pattern production rates over test segments on the C-DF, since all patterns required four lines for completion.

The original design fluency tests were conceived to provide a non-verbal analogue of verbal fluency tests [9]. However, practical considerations resulted in the use of response sheets either for the entire test (on the 5-PT), or for each test condition. For example, five response sheets are used (each for one minute of pattern generation) in the RFFT. As a result, previous patterns remain visible as the test progresses, and can be used as cues for generating subsequent patterns and avoiding repetitions. In contrast, during the C-DF each pattern disappears after being drawn. As a result, participants need to remember previous patterns to avoid repetitions. We therefore anticipated a higher relative incidence of repetitions on the C-DF than on other design fluency tests.

In Experiment 1, we examined the influence of sex, age, and education on C-DF performance in a normative population of 180 participants ranging in age from 18 to 82 years. We anticipated that familiarity with computers might influence C-DF performance because participants familiar with computers would also have greater familiarity in manipulating the mouse. Therefore, we also analyzed the influence of computer-use on performance.

In addition, we analyzed correlations between design fluency scores and scores on other computerized neuropsychological tests administered on the same day. Previous studies have generally found small but significant correlations between design fluency scores and scores on processing speed tests such as the Trail Making Test [3,6,20], as well as significant correlations with verbal fluency test performance [3,12,20–22] and performance IQ [9].

### General methods

#### Ethics statement

Participants in all experiments gave informed written consent following procedures approved by the Institutional Review Board of the VA Northern California Health Care System (VANCHCS) and were paid for their participation.

#### Participants

We tested 180 control subjects (mean age = 41.6 years, range 18 to 82 years) whose demographic characteristics are summarized in Table 2. Participants were recruited from advertisements on Craigslist (sfbay.craigslist.org) and pre-existing control populations. The participants were highly educated, with an average of 14.5 years of education, and 59% were male. The participants were required to meet the following inclusion criteria: (a) fluency in the English language; (b) no current or prior history of psychiatric illness; (c) no current substance abuse; (d) no concurrent history of neurologic disease known to affect cognitive functioning; (e) on a stable dosage of any required medication; (f) auditory functioning sufficient to understanding normal conversational speech and (g) visual acuity normal or corrected to 20/40 or better. Subject ethnicities were 64% Caucasian, 12% African American, 14% Asian, 10% Hispanic/Latino, 2% Hawaiian/Pacific Islander, 2% American Indian/Alaskan Native, and 4% “other.” Subjects indicated the daily hours of computer-use on a separate questionnaire containing an 8-point Likert scale, with the options of “1: Never; 2: Less than 1 hour per week; 3: Less than 1 hour per day; 4: 1–2 hours per day; 5: 2–3 hours per day; 6: 3–4 hours per day; 7: 4–6 hours per day; 8: More than 6 hours per day”.

**Table 2:** Participant characteristics.

**Experiment**	**Group**	**N**	**Ages (yrs)**	**Education (yrs)**	**C-use scale**	**Male (%)**
**Exp. 1**	Control/ Normative	180	18–82; 41.6 (21.4)	10–20; 14.6(2.1)	5.09	59%
**Exp. 2/3**	Control/ Malinger	55/52	18–46; 26.2 (5.5)	12–18; 14.8 (1.4)	5.86	51%
**Exp.4**	mTBI	24	20–61; 34.1(11.5)	10–18; 13.5(1.4)	5.04	100%
**Experiment**	**Group**	**N**	**Ages (yrs)**	**Education (yrs)**	**C-use scale**	**Male (%)**
**Exp. 1**	Control/ Normative	180	18–82; 41.6 (21.4)	10–20; 14.6(2.1)	5.09	59%
**Exp. 2/3**	Control/ Malinger	55/52	18–46; 26.2 (5.5)	12–18; 14.8 (1.4)	5.86	51%
**Exp.4**	mTBI	24	20–61; 34.1(11.5)	10–18; 13.5(1.4)	5.04	100%
	sTBI	4	35–52;45.0(7.1)	12–16; 13.0(1.2)	5.25	75%

C-use = computer-use scale; mTBI = mild TBI. sTBI = severe TBI. Range, mean, and variance are shown for age and education.

Design Fluency was the tenth test in the California Cognitive Assessment Battery (CCAB), which included measures of performance on finger tapping [23,24], simple reaction time [25,26], Stroop, digit span forward and backward [27,28], phonemic and semantic verbal fluency, verbal list learning, spatial span [29,30], trail making [31], vocabulary, the Wechsler Test of Adult Reading (WTAR), choice reaction time [32,33], risk and loss avoidance, delay discounting, the Paced Auditory Serial Addition Task (PASAT), the Cognitive Failures Questionnaire (CFQ), the Posttraumatic Stress Disorder Checklist (PCL) [34], and a local demographic and medical information questionnaire that included the computer-use question. Testing was performed in a quiet room using a standard Personal Computer (PC) controlled by Presentation® software (Versions 13 and 14, NeuroBehavioral Systems, Berkeley CA). The C-DF required 2–4 minutes for completion. An executable, open-source version of the C-DF test is available for download at http://www.ebire.org/hcnlab/ and an Excel spreadsheet with the data from the experiments described below can be downloaded at https://figshare.com/articles/Data_from_CCAB_computerized_design_fluency_test/3115120.

**Fig 1:**
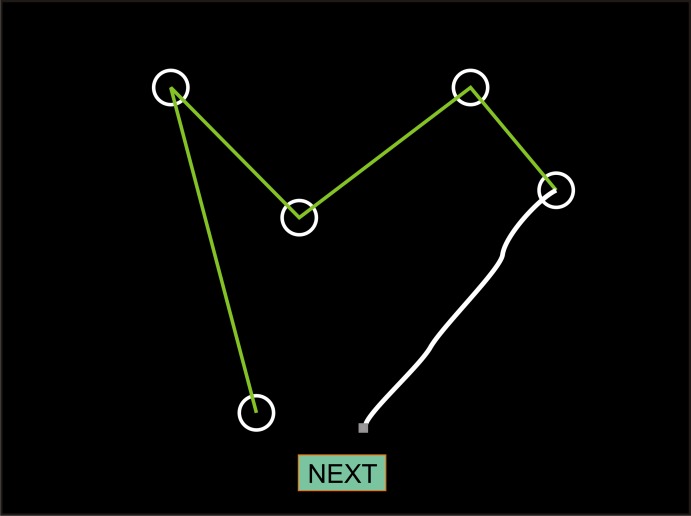
The design fluency test. Participants connected circles on the display with four lines drawn with the mouse. As each line was drawn, the path was shown in white. When the cursor crossed a circle, it was included in the figure and connected with the previously selected circle by a straight green line. When a design was finished, participants moved the cursor (small gray square) to click the “NEXT” box to advance to the next trial.

Fig 1 shows the paradigm. Subjects sat approximately 0.5 m from a 17” Samsung Syncmaster monitor (refresh rate = 60 Hz) and viewed a display of five white circles (10.5 mm diameter, 2^0^ visual angle) and a green “Next” box on a black background. A small, gray square was used as a cursor controlled by the mouse (mouse sensitivity was set at the mid-point of the mouse sensitivity scale). Participants were instructed to use the mouse to draw as many connected 4-line patterns as possible, while avoiding repetitions, during the 90 s test. Participants did not see the countdown timer during the test.

When the cursor was in a circle, the circle’s color changed to green to indicate that it had been selected. After selection, the circle color reverted to white and the cursor’s path (previously shown as a white line drawn by the participant) was replaced by a straight green line connecting the circles. When one pattern was finished, the participant clicked the “Next” button at the bottom of the screen to proceed to the next trial. The screen was then cleared and the cursor was positioned above the “Next” button to begin the subsequent trial.

Before practice trials began, participants watched the computer draw an example of a correct design. Participants then performed a series of practice trials using a simplified circle array. Practice was not time-limited, and visual feedback was provided after each trial, indicating the type of error that occurred, if any. Practice terminated after three correct (not necessarily consecutive) practice trials.

After the practice trials, the display cleared and participants were reminded to draw as many designs as possible without repeating patterns during the test period. Test onset was cued with “Get Ready” on the monitor, followed after 2.0 s by “Go Go Go!” which was followed after 1.0 s by the appearance of the five circles on the screen. Timing began with the appearance of the circles. Participants were required to produce continuous 4-line patterns that could be open (connecting five circles) or closed (connecting four circles). After 90 s, the display cleared and the text “Time’s Up” appeared on the screen, indicating the completion of the test. Any partially completed designs were excluded from scoring.

#### Scoring

The time of selection was recorded for all circles selected. Trials in which fewer than four lines were drawn occurred infrequently (1.6% of trials) and were scored as incorrect, and trials with more than four lines (7.6% of trials) were truncated to the first four lines because participants sometimes inadvertently crossed an extra circle as they were moving the cursor to the “NEXT” button. In order to determine if repetitions had occurred, the first four lines of each design were used to generate an eight-digit sequence code that was independent of the order or direction in which the lines had been drawn. These sequence codes were compared to identify repetitions.

#### Statistical analysis

The results were analyzed with Analysis of Variance (ANOVA) using CLEAVE (www.ebire.org/hcnlab). Greenhouse-Geisser corrections of degrees of freedom were uniformly used in computing p values in order to correct for covariation among factors and interactions, with effect sizes reported as partial ω^2^. Pearson correlations were used to describe relationships between measures and to identify demographic factors (e.g., age and education) that significantly influenced performance, with significance levels evaluated with Student’s t-tests. Multiple linear regression analysis was used to further identify demographic factors with independent influences on performance and to correct for their contributions in order to generate z-scores.

### Results: Experiment 1

**Fig 2:**
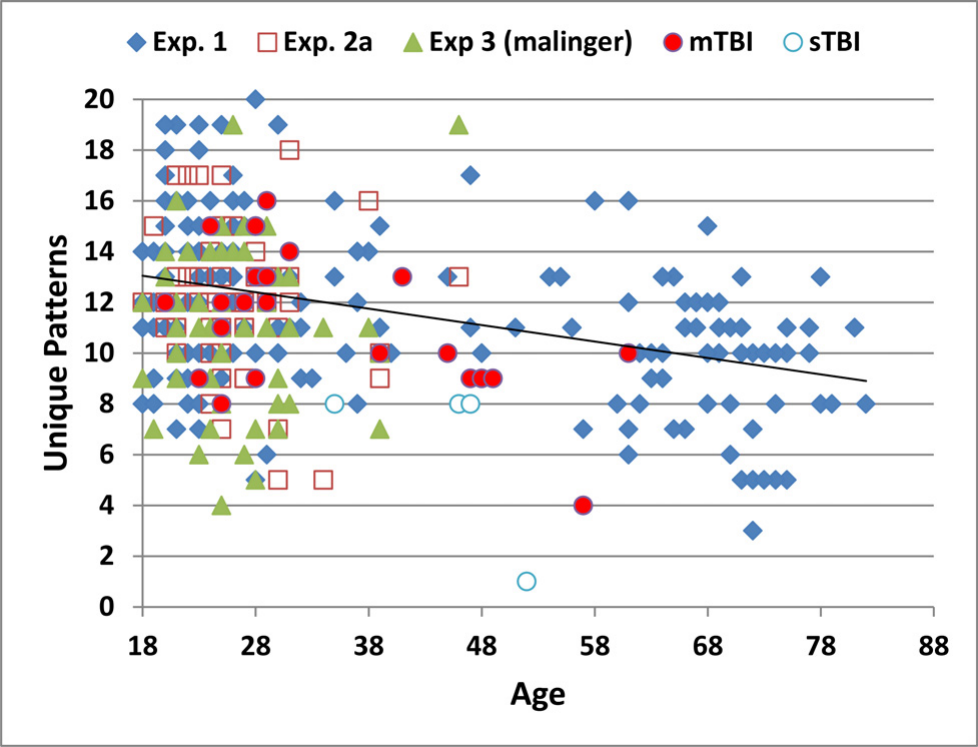
The number of unique patterns produced in 90s as a function of participant age. The age-regression slope from Experiment 1 (blue diamonds) is shown, along with the results from the first test session of Experiment 2 (2a, open red squares), Experiment 3 (simulated malingering, green triangles), and Experiment 4 shown separately for patients with mild TBI (mTBI, filled red circles), and severe TBI (sTBI, striped red circles).

Fig 2 shows the number of unique patterns produced as a function of age in Experiment 1 (blue diamonds) and in the other experiments (discussed below). Table 3 provides a summary of performance measures for the different experiments. The participants in Experiment 1 produced 11.54 unique patterns (range 3–20) over the 90 s test period, including 8.39 patterns over the first 60 s. The rate of pattern production was considerably lower than that observed with the 5-PT and RFFT, and somewhat lower than that seen with the Delis-Kaplan Executive Function System (D-KEFS), which, like the C-DF, requires that each pattern includes four lines (see Table 1). The CV (28.6%) of the C-DF unique pattern score was similar to the CVs of most other design fluency tests (see Table 1).

**Table 3:** Mean results from the four experiments.

	Type	N	UPs	RPs	% RPs	UP z-score
Exp. 1	Norm	183	11.54 (3.29)	1.61 (1.42)	11.41(9.30)	0.00 (1.00)
Exp. 2a	Control	55	11.98 (2.83)	1.45 (1.37)	9.96 (9.35)	-0.29 (1.06)
Exp. 2b	Control	55	12.62 (2.64)	1.36 (1.38)	9.42 (9.35)	-0.06 (1.07)
Exp. 2c	Control	55	13.62 (2.38)	1.65 (1.32)	10.48 (7.75)	0.30 (0.92)
Exp. 3	Malinger	52	10.88 (3.43)	2.48 (2.19)	17.82 (13.82)	-0.71 (1.33)
Exp. 4	mTBI	24	11.46 (2.87)	1.67 (1.99)	11.35(11.73)	-0.15 (1.03)
Exp. 4	sTBI	4	6.25(3.50)	1.00 (0.82)	10.56 (8.19)	-1.89 (1.52)

Standard deviations are shown in parentheses. Z-score = unique pattern z-score corrected for age and computer-use. See Table 1 for additional abbreviations.

Participants produced relatively few repeated patterns (1.61, sd = 1.42, range 0–7). Repetition scores showed substantial intersubject variability (CV = 88.1%): 54% of participants produced fewer than two repetitions, while 11% produced four or more.

Table 4 shows the influence of demographic factors on performance. Age, education, and computer-use correlated significantly with the number of unique patterns produced. As in previous studies [3,6,9,11], there was a substantial decline in unique pattern production with age [r = -0.42, t(178) = - 6.17, p < 0.0001]. Years of education was only marginally correlated with unique pattern production [r = 0.14, t(178) = 1.89, p < 0.07]. However, our test group included many students who were still in college, so age was positively correlated with educational attainment [r = 0.19, t(178) = 2.58, p < 0.02]. Multiple regression analysis with both Age and Education as factors showed that these factors conjointly accounted for 30.1% of variance, with both showing significant influence on unique pattern scores [Age, t(177) = -6.88, p < 0.0001, Education t(177) = 3.34, p = 0.001]. Consistent with previous results [6,10], there were no significant differences in the scores of male and female participants [r = 0.01, NS].

**Table 4:** Demographic factors influencing performance.

	Edu	C-use	Sex	UPs	RPs	%RPs	UP z-score
**Age**	**0.19**	**-0.24**	**0.09**	**-0.42**	**-0.19**	**-0.08**	**0.00**
**Edu**		**0.33**	**0.09**	**0.14**	**0.00**	**-0.02**	**0.10**
**C-use**			**0.00**	**0.44**	**0.13**	**0.02**	**0.00**
**Sex**				**0.01**	**0.12**	**0.13**	**0.05**
**UPs**					**0.21**	**-0.04**	**0.84**
**RPs**						**0.94**	**0.12**
**%RPs**							**-0.06**

Pearson product-moment correlations between demographic and performance measures in the 180 participants of Experiment 1. Given the sample size, significance levels (uncorrected for multiple comparisons) are |r| > 0.15, p < 0.05; |r| > 0.19, p < 0.01; |r| > 0.25, p < 0.001; and |r| > 0.29, p < 0.0001. See Tables 1 and 2 for additional abbreviations.

A strong correlation was also seen between unique pattern scores and computer-use [r = 0.44 t(178) = 6.44, p < 0.0001]. Indeed, the influence of computer-use was significantly greater than that of education [z = 2.72, p < 0.01]. Multiple regression with Age, Education, and Computer-use as factors accounted for 31.0% of score variance and revealed that both Age [t(176) = -5.35, p < 0.0001] and Computer-use [t(176) = 4.63, p < 0.0001] had significant influences, whereas the influence of Education fell to insignificance [t(176) = 1.42, p < 0.20].

**Fig 3:**
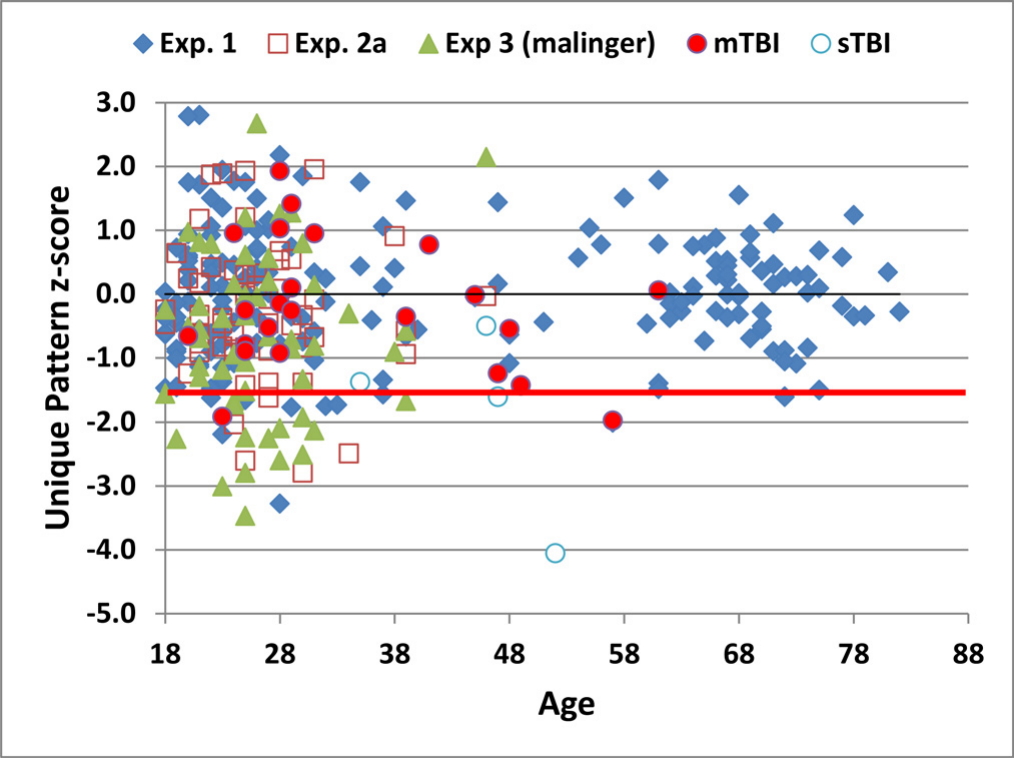
Unique pattern z-scores as a function of age. Z-scores showing the number of unique patterns produced from participants as a function of age after correction for the effects of age and computer use. The red line shows the p<0.05 abnormality threshold as defined in Experiment 1.

Raw scores were therefore transformed into z-scores using the following age- and computer-use regression function, Score = 10.5 + 0.62*computer-use– 0.051*Age. Age- and computer-use accounted for 30.2% of the variance in unique pattern scores, resulting in a reduced standard deviation in the transformed data, with a CV (23.8%) that was similar to the CVs obtained in age and education stratified norms on the RFFT [9]. The resulting z-score distribution, reflecting the difference between observed and predicted scores, is shown as a function of age in Fig 3.

**Fig 4:**
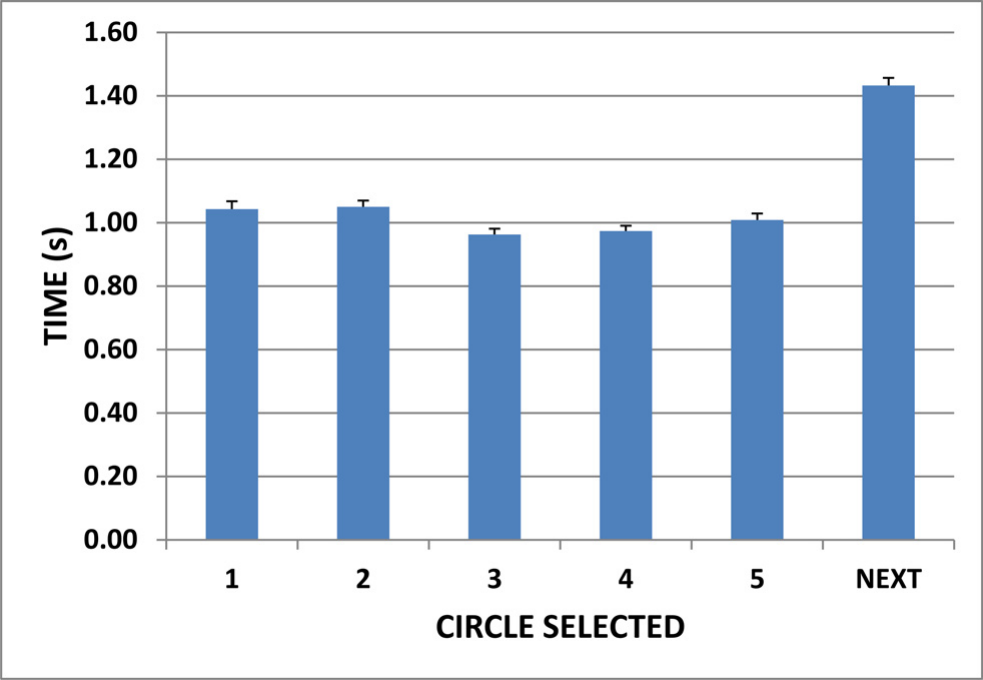
Mean item-selection latencies The latency of selection for circle 1 was measured from the beginning of the display, and latencies of each subsequent circle and the “NEXT” response were measured relative to the selection of the previous circle. Error bars show 95% confidence intervals. Data from successive correct 4-line patterns in Experiment 1.

We also analyzed the timecourse of pattern completion. The mean response latencies associated with the selection of each of the five circles and the “Next” button are shown in Fig 4. The selection of each circle required about 1.0 s, and the selection of the “Next” button required an additional 1.4 s, so that overall mean trial completion for a 4-line pattern required approximately 6.5 s.

**Fig 5:**
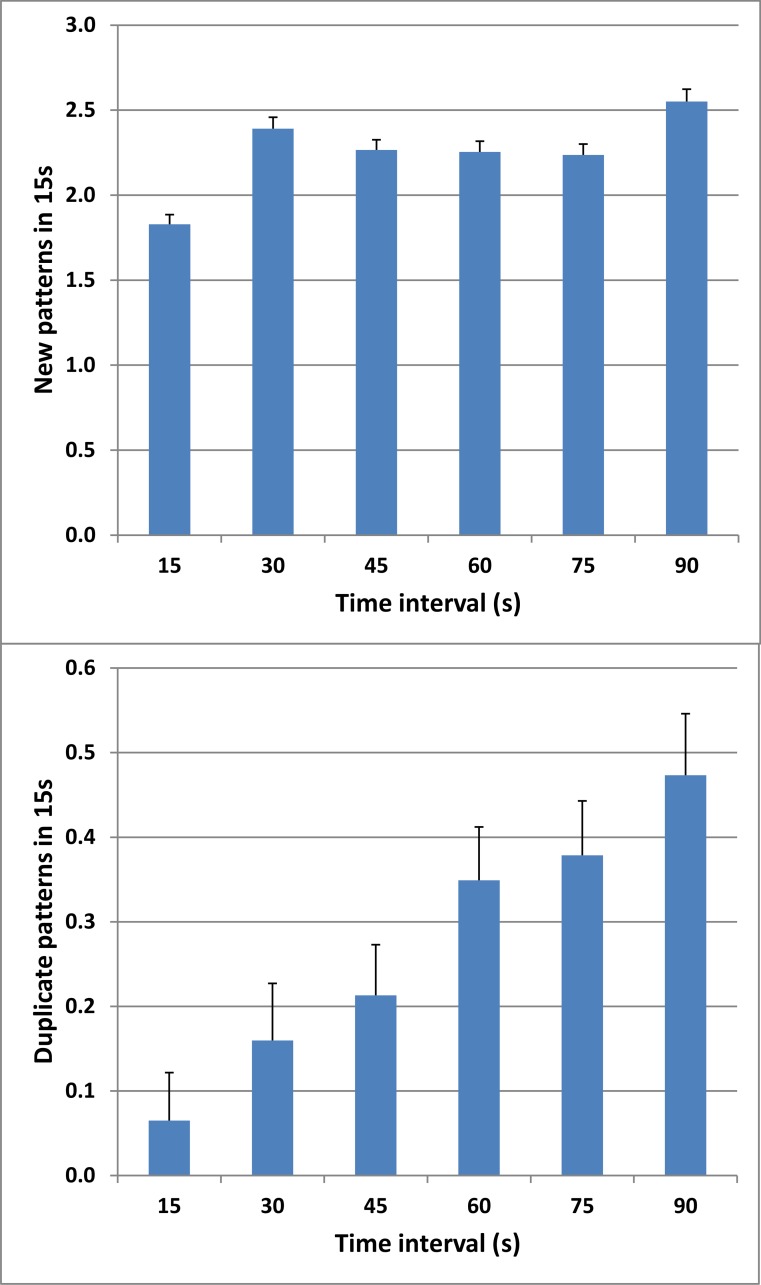
Pattern production rate. The number of unique patterns (top) and repeated patterns (bottom) produced over successive 15 s intervals by the participants in Experiment 1. Error bars show standard errors of the mean.

Fig 5 (top) shows the rate of pattern production over successive 15 s periods. Unlike other design fluency tests, fewer patterns were produced during the first 15 s of the C-DF than during subsequent epochs. ANOVA for repeated measures showed small but significant variations in the number of patterns produced across the 15 s periods [F(4,684) = 27.52, p < 0.0001, partial ω^2^ = 0.13].

Repetitions constituted 12.3% of all patterns. Their timecourse is shown in Fig 5 (bottom). There was a weak positive correlation between the number of unique patterns and the number of repetitions [r = 0.21, t(178) = 2.87, p < 0.005], but no significant correlation between unique pattern scores and the percentage of repetitions [r = -0.04, NS]. The percentage of repetitions did not correlate with age [r = -0.08, NS], education [r = -0.02, NS], or computer-use [r = 0.02, NS]. Fig 5 (bottom) shows the number of repetitions in each successive 15 s interval. As in previous reports [8], the incidence of repetitions increased throughout the test [F(4,684) = 116.59, p < 0.0001, partial ω^2^ = 0.40], with repetitions constituting more than 18% of patterns produced during the final 75–90 s interval.

Table 5 shows the correlations between C-DF unique pattern z-scores and z-scores on other computerized neuropsychological tests. C-DF z-scores showed small but significant negative correlations with z-scores on processing speed tests including Trails A (-0.21), Trails B (r = -0.22) [31], choice reaction time (-0.28) [32], and question completion time (-0.20) [34], and small positive correlations with z-scores on tests of verbal fluency (0.18) [35], spatial span (0.22) [30], and digit span (0.17) [27]. In contrast, the percentage of repetitions showed marginally significant negative correlations only with spatial span measures (-0.15) and question completion time (-0.18).

**Table 5:** Correlations with z-scores from other neuropsychological tests.

	Trails A	Trails B	Verbal fluency	Spatial span	Choice reaction time (CRT)	Simple reaction time	Digit span	Question completiontime
z-score	-0.21	-0.22	0.18	0.22	-0.28	-0.22	0.17	-0.20
%RPs	-0.09	0.05	0.10	-0.15	-0.02	-0.05	-0.11	-0.18

Correlations of Experiment 1 unique pattern z-scores and percentage of repeated patterns with z-scores from other computerized neuropsychological tests. Verbal fluency z-scores were combined across semantic (animal) and phonemic (“F”) conditions. See Table 1 for abbreviations. Correlations exceeding |r| = 0.15 (one tailed) are significant at p<0.05 (uncorrected for multiple comparisons).

#### Discussion: Experiment 1

Participants produced fewer unique patterns in the C-DF than in other design fluency tests: 11.5 unique patterns over the full 90 s test period and 8.4 unique patterns over the first 60 s in contrast to the 15.0 patterns in the first 60 s by the participants in Santa Maria et al. [8] and the 17.3 patterns produced by the participants in the RFFT normative sample [9]. Three factors are likely responsible for the reduced unique pattern production in the C-DF: (1) Participants were required to include four lines in each pattern, whereas 1-, 2-, and 3-line patterns are permissible in the 5-PT and RFFT. An analysis of drawing times (Fig 4) shows that each line adds approximately one second to pattern completion time; i.e., drawing a 1-line pattern would require roughly three seconds less than drawing a 4-line pattern. Thus, fewer patterns would be expected on 4-line design fluency tests like the C-DF and D-KEFS than on tests, like the 5-PT and RFFT, where 1-line patterns are acceptable (see Table 1). (2) The C-DF test required participants to click the NEXT button after completing each drawing. This additional response added approximately 20% to overall pattern completion time (see Fig 4). Eliminating the delay associated with the “NEXT” response would have resulted in an estimated 10.8 patterns during the first 60s of the C-DF, i.e., a production rate similar to that seen in the D-KEFS design fluency test [14–16]. (3 The pattern production rate on the C-DF was likely reduced because drawing with a mouse is less familiar and natural than drawing with a pen and paper.

An expected regular increase in the incidence of repetitions over time was observed (Fig 5, bottom), reflecting the fact that the probability of repetitions increased with number of unique patterns previously produced. The increased percentage of repeated patterns (12.2%) compared to most previous studies (see Table 1) may reflect memory lapses, since the participants in the C-DF test depended on memory rather than on the visual inspection of previously produced patterns to avoid repetitions.

#### Demographic effects

We found a negative correlation between age and unique pattern scores that was similar to the correlations reported in previous studies [3,6,9,10,20,22]. The age slope was relatively steep [9,10], so that the mean number of patterns produced by the oldest participants was approximately one standard deviation below the number produced by the youngest participants.

As in previous studies, [3,6,9,10], increased education was associated with improved performance. However, we found that computer-use had a more significant influence on performance than did education. We have also found stronger performance correlations with daily computer-use than with education in other computerized tests that require mouse manipulation [26,30–32,34]. The stronger correlation between computer-use and performance than between education and performance may have reflected a confounding interaction with age: we found a negative correlation between age and computer use (r = -0.24), whereas age was positively correlated with education (r = 0.19).

#### Correlations with other neuropsychological tests

The correlations between performance on the C-DF and other neuropsychological tests were similar to those previously reported. For example, previous studies have found significant correlations between design fluency scores and performance on Trail Making Test [3,6,20] and verbal fluency [3,12,20–22] tests. However, the magnitude of correlations that we found were predictably lower than those reported in previous studies due to the fact that we correlated z-score measures (corrected for the influence of age, education, and computer-use), rather than raw scores.

#### Pattern production rate

In contrast to previous studies reporting significant declines over time in the rate of pattern generation on the 5-PT [4,5,8], we found that the production rate on the C-DF showed a small increase after the initial 15 s. One likely explanation is that participants in the 5-PT begin with one- and two-line patterns that can be rapidly drawn. Since these patterns are relatively rare (about 5% of all possible patterns), participants switch to 3- and 4-line patterns later in the test, reducing the rate of pattern generation. In contrast, we found that the rate of pattern generation on the C-DF increased after the first 15 s period, perhaps reflecting familiarization with the circle display positions and mouse control parameters.

#### Measurement sensitivity

The CV of C-DF unique pattern scores was similar to the CVs obtained on manually administered tests, while the reduced CV seen for age- and computer-use regressed scores was similar to the CVs of age- and education-stratified norms on other design fluency tests [9]. As in previous DF tests [3], the percentage of repeated patterns showed high variance, limiting its potential clinical utility.

## Experiment 2: Generalization, Test-Retest Reliability and Learning Effects

In Experiment 2, 55 young control participants underwent three successive test sessions at weekly intervals. This allowed us to evaluate the goodness of fit of the regression functions derived from the normative data in Experiment 1 to a younger and better-educated control population. In addition, repeated testing permitted the quantification of C-DF test-retest reliability and learning effects. Previous design fluency studies have found high test-retest reliability and significant performance improvements over repeated tests [5,6,9,11,12] along with poor test-retest reliability for number of repeated patterns (often termed perseverative errors) [10].

### Methods: Experiment 2

#### Participants

The demographic characteristics of the participants in Experiment 2 are shown in Table 2. The 55 young volunteers (mean age = 26.2 years) were recruited from internet advertisements on Craigslist (sfbay.craigslist.org). The group was very well-educated (average of 14.8 years of education), with many of the younger participants still enrolled in college. Fifty-one percent were male. Ethnically, 68% were Caucasian, 11% Latino, 9% African American, 10% Asian, and 2% other. The participants were required to meet the same inclusion criteria listed in Experiment 1. Participants underwent three CCAB test sessions at approximately weekly intervals.

#### Statistical analysis

Analysis of Variance (ANOVA) for repeated measures was used to evaluate learning effects. Test-retest reliability was evaluated with intraclass correlation coefficients (ICCs) calculated with SPSS (IBM, version 22).

### Results: Experiment 2

Mean performance metrics from the three test sessions of Experiment 2 are included in Table 3. The performance of individual participants in the first test session is shown in Fig 2 (2a, open red squares). The z-scores from participants in Experiment 2, adjusted for age and computer-use using the regression functions from Experiment 1, are included in Fig 3. Participants in Experiment 2a produced an average of 11.98 (2.83) patterns (range 5 to 18) with a CV of 23.6%. In the first test session of Experiment 2, neither the unique pattern z-score (-0.29) nor the percentage of pattern repetitions (10.8%) differed significantly from those in Experiment 1 [F(1,233) = 3.48, p <0.07, and F(1,233) = 1.02, NS, respectively].

**Fig 6:**
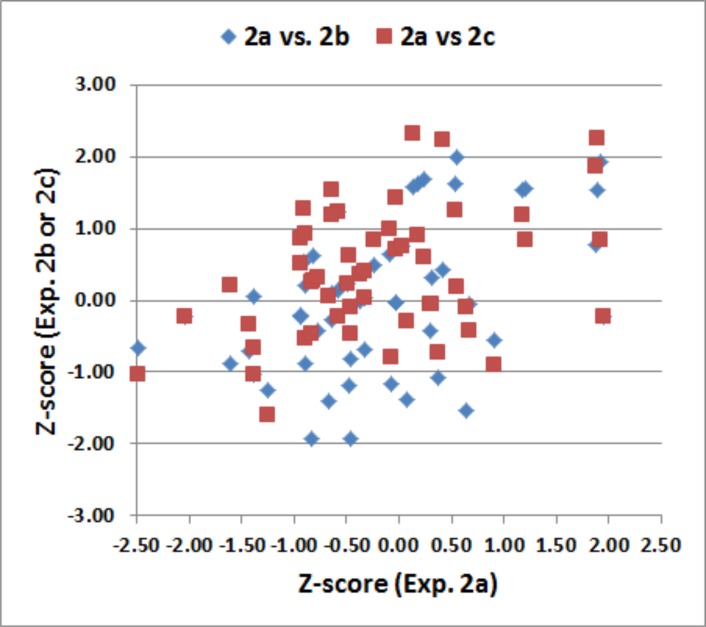
Test-retest reliability. Z-scores from each subject in Experiment 2a are shown plotted against z-scores from Experiment 2b (blue diamonds) and Experiment 2c (red squares). Pearson correlations were 0.53 between 2a and 2b, 0.48 between 2a and 2c, and 0.66 between 2b and 2c. The overall intraclass correlation coefficient was 0.79.

Fig 6 shows scatter plots of the number of unique patterns produced by each participant in the three test sessions of Experiment 2. We found an ICC of 0.79 for the unique pattern z-scores over the three test sessions, and an ICC of 0.55 for the percentage of repeated patterns.

As expected, the number of unique patterns increased significantly over repeated tests (by 13.7%) [F(2,108) = 10.59, p < 0.001, partial ω^2^ = 0.15]. In contrast, the percentage of repetitions did not change significantly [F(1,233) = 0.28, NS].

### Discussion: Experiment 2

The z-scores of participants in Experiment 2 did not differ significantly from those in Experiment 1, indicating that the normative data obtained in Experiment 1 generalized to the different normative sample of younger and slightly better educated participants in Experiment 2.

The ICC that we obtained (0.79) was similar to the ICCs (range 0.76 to 0.84) found in previous design fluency studies [3,6,9]. The ICC of pattern repetitions was somewhat higher than in previous studies [3,6,9], but still showed reduced reliability overall.

The learning effects seen across repeated tests were similar in magnitude to those observed in repeated testing with manually administered design fluency tests [3,9]. These effects have been reported to persist at inter-test intervals of one or more years [11,36].

## Experiment 3: Effects of Simulated Malingering

Identifying participants with invalid performance can be a significant challenge in neuropsychological testing, particularly among patients with mild head injury [37]. Among such patients who have litigation or pension claims, a high percentage show evidence of malingering on performance-validity tests [38,39]. However, only one previous study has investigated the effects of malingering on DF tests: Demakis [40] found significant performance deficits in undergraduates instructed to simulate malingering on the RFFT. On average, malingerers produced unique pattern scores 1.63 standard deviations below those produced by control participants performing in full-effort conditions.

### Methods: Experiment 3

#### Participants

The participants were identical to those of Experiment 2, except that three of the 55 participants failed to return for the malingering test session.

#### Materials and procedures

The methods and procedures were identical to those of Experiments 1 and 2a, but participants were given additional instructions. After the third session of Experiment 2, participants were given written instructions to feign the symptoms of a patient with mild TBI during a fourth test session that included all of the CCAB tests during the following week. The instructions were as follows: “Listed below you’ll find some of the symptoms common after minor head injuries. Please study the list below and develop a plan to fake some of the impairments typical of head injury when you take the test. Do your best to make your deficit look realistic. If you make too many obvious mistakes, we’ll know you’re faking! Symptom list: Difficulty concentrating for long periods of time, easily distracted by unimportant things, headaches and fatigue (feeling “mentally exhausted”), trouble coming up with the right word, poor memory, difficulty performing complicated tasks, easily tired, repeating things several times without realizing it, slow reaction times, trouble focusing on two things at once.”

#### Statistical analysis

The results were analyzed using Analysis of Variance (ANOVA) between groups to compare the results with those of the normative controls in Experiment 1, and ANOVA within groups to compare the results to those in Experiment 2a. Other procedures were identical to those of Experiment 1.

### Results: Experiment 3

Mean performance measures from Experiment 3 are included in Table 3, with data from individual simulated malingerers included in Figs 2 and 3 (green triangles). Simulated malingerers showed significantly lower z-scores (-0.71) than the normative controls in Experiment 1 [F(1,228) = 17.04, p < 0.0001, partial ω^2^ = 0.07]. Overall, 26.9% of malingering participants produced z-scores in the abnormal range (p < 0.05, z-score < -1.57), and 9.6% produced scores in the extremely abnormal range (p<0.005, z-score < -2.31). Surprisingly, z-scores did not differ significantly from those obtained by the same subjects in Experiment 2a [F(1,51) = 1.59, NS].

Simulated malingerers also showed a higher percentage of repetitions than the normative controls in both Experiment 1 [F(1,228) = 14.96, p < 0.0003, partial ω^2^ = 0.06] and Experiment 2a [F(1,51) = 16.37, p < 0.0002, partial ω^2^ = 0.24]. Half of the malingerers with abnormal unique pattern scores also showed abnormal (p < 0.05) repetition percentages. In addition, 13.4% of simulated malingerers with unique pattern z-scores in the normal range showed an abnormal incidence of repetitions.

### Discussion: Experiment 3

The results of Experiment 3 were consistent with those of Demakis [40]: simulated malingerers produced fewer patterns and a higher incidence of repetitions than control participants. Nevertheless, the effects of simulated malingering were relatively modest, and even failed to reach statistical significance when compared to the performance of the same subjects in their first full-effort condition (i.e., Experiment 2a). As a result, at the criterion needed to provide 95% specificity, sensitivity was only 26.9%. At stricter criterion levels, specificity improved, but was accompanied by steep declines in sensitivity.

Overall, C-DF performance was less sensitive to simulated malingering than performance on other computerized tests. For example, simple reaction time measures showed a sensitivity of 83% and a specificity of 100% in classifying control participants and simulated malingerers [25]. We have argued elsewhere that malingering effects are reduced when tasks become more complex and engaging [33]. For example, malingering effects are reduced in choice reaction time relative to simple reaction time [33], and on the Trail Making Test part B relative to the Trail Making Test part A [31]. In Experiment 3, the C-DF was a complex task that required considerable attentional engagement, possibly limiting the scope of malingering.

#### Limitations

The magnitude of the malingering effect (z-score = -0.71) was smaller than that of Demakis [40] on the RFFT (estimated mean z-score -1.63). One possible explanation is that the participants in Experiment 3 had improved their “baseline” performance through repeated exposure to the C-DF test in Experiment 2. Insofar as the participants in Experiment 3 adjusted their malingering performance relative to their performance baseline in Experiment 2c, the magnitude of their malingering effect would have been reduced by 0.59 z-scores due to learning.

In addition, the relatively high standard deviation (2.75) even of regressed unique pattern scores meant that their unique pattern scores would need to be very small to fall clearly outside the range of normal variability. For example, for a malingering subject to produce a z-score of -2.5, they would have needed to produce fewer than 6.0 unique patterns, rather than the 10.9 unique patterns that they actually produced. Many simulated malingerers may have felt that such a low rate of unique pattern production would have been easily detectable by the examiner.

## Experiment 4: Effects of Traumatic Brain Injury

Previous studies have shown that design fluency performance is impaired after brain lesions, particularly lesions affecting the right frontal lobe [1,41,42]. A number of studies have also found reduced scores in patients with severe TBI (sTBI) [43–45], along with deficits in patients with mild TBI (mTBI) when tested in the acute phase [46]. However, other studies have found that the D-KEFS design fluency tests have limited sensitivity in discriminating TBI patients with documented lesions from controls [47]. In Experiment 4, we evaluated the sensitivity of the C-DF in a small cohort of veterans with varying histories of mild and severe combat-related TBI.

### Methods: Experiment 4

#### Participants

Thirty Veterans with a diagnosis of TBI made after comprehensive neurological and neuropsychological examination were recruited from among the Veterans Affairs Northern California Health Care System (VANCHCS) patient population. Two mTBI patients had shown evidence of invalid performance on other computerized tests [24,25,31,33] and produced low C-DF z-scores in the current test (-1.01 and -2.33). Their data were therefore excluded from further analysis.

The remaining patients included 27 males and one female between the ages of 20 and 61 years (mean age = 35.8 years), with an average 13.4 years of education (Table 2). All patients had suffered head injuries and transient loss or alteration of consciousness, and had been medically diagnosed as suffering from TBI after neurological and neuropsychological examination. Most were recent combat veterans, including 24 patients who had suffered one or more combat-related incidents with a loss of consciousness of less than 30 minutes, no hospitalization, and no evidence of brain lesions on clinical MRI scans. These patients were categorized as mTBI.

The remaining four patients had histories of severe accidents with hospitalization, brain abnormalities visible on neuroimaging, coma durations exceeding eight hours, and post-traumatic amnesia exceeding 72 hours. These patients were categorized as sTBI. The patients were informed that the study was for research purposes only and that the results would not be included in their official medical records. All patients were tested at least one year post-injury.

Evidence of posttraumatic stress disorder (PTSD), as reflected in elevated scores on the PTSD Checklist (PCL), was evident in the majority of the TBI sample. Additional information about the severity and etiology of the TBIs is included in S1 Table.

#### Materials and procedures

The methods were identical to those of Experiment 1.

#### Statistical analysis

The results were compared to those of the Experiment 1 normative population using the age- and computer-use regression functions established in Experiment 1.

### Results: Experiment 4

Mean performance measures from mTBI and sTBI patients are included in Table 3, with the data from individual patients included in Figs 2 and 3, shown separately for mTBI (filled red circles) and sTBI (striped red circles). There was no significant correlation between scores on the PCL checklist and C-DF performance [r = -0.06, NS]. ANOVA showed that the Group effect (Experiment 1 controls, mTBI, sTBI) was significant [F(2,205) = 6.93, p < 0.002, partial ω^2^ = 0.05]. Subsequent analyses showed insignificant differences between mTBI patients and controls [F(1,202) = 0.50, NS], but a significant difference between sTBI patients and controls [F(1,202) = 13.66, p < 0.0003, partial ω^2^ = 0.06], as well as significant differences between sTBI patients and mTBI patients [F(1,26) = 8.53, p < 0.01, partial ω^2^ = 0.22]. In contrast, there was no significant Group effect on the percentage of repetitions [F(2,205) = 0.02, NS].

### Discussion: Experiment 4

These results are consistent with previous reports of impaired design fluency performance in patients with sTBI [43,48]. Ruff et al. [43] found more severe deficits in sTBI patients than in patients with moderate TBI, but did not test patients with mTBI. We found no significant differences between the mTBI and control populations, suggesting that post-TBI deficits in the chronic phase are largely restricted to patients with sTBI. This absence of significant C-DF deficits in patients with mTBI is consistent with generally good recovery of cognitive performance even in mTBI patients with blast-related TBI [49].

#### Limitations

These results should only be considered preliminary given the small sample size, particularly of the sTBI patient population.

## General Discussion

The properties of the C-DF test resemble those of manually administered DF tests. C-DF performance declined with age and improved with increasing education and computer experience. Unique pattern z-scores showed modest but significant correlations with z-scores on tests of processing speed, memory, and executive function. The CV was similar to that of manually administered DF tests, and the use of age- and computer-use regression functions reduced CVs to levels similar to those of manually administered DF tests when participants are stratified by age and education. The test-retest reliability of the C-DF was high, and learning effects across repeated test sessions were comparable to those of manually administered tests. In addition, the C-DF showed performance impairments in patients with severe TBI that were similar to those reported for manually administered DF tests.

However, the C-DF test has a number of desirable properties in comparison with manually administered DF tests. (1) Administration is automated, reducing the influence of the examiner on test results and standardizing test administration procedures across different laboratories. We found that C-DF norms and regression functions established in Experiment 1 generalized to younger control participants (Experiment 2a), although further normative studies with larger and more varied populations would be necessary before widespread clinical use. (2) C-DF scoring is automatic and error-free, facilitating test interpretation. (3) A complete set of test results, including the timing of individual responses, is automatically obtained. This facilitates the analysis of performance changes over time and eliminates the need for the archival storage of paper records.

It should be noted that the profile of clinical sensitivity of the C-DF, where patients must remember previously produced patterns, may differ from that of existing design fluency tests, where patients can see the patterns that have been previously produced on the scoring sheet. As a result, the C-DF would be expected to place greater demands on storage and retrieval from visuospatial memory, whereas other existing tests may place greater demands on visual scanning and executive strategies for generating pattern variants.

Finally, we did not investigate whether variations in computer hardware (e.g., monitor size, mouse gain settings, etc.) influenced C-DF performance. We did find that performance was significantly influenced by computer experience, presumably reflecting familiarity with using the mouse to draw lines. Further studies are underway to evaluate C-DF performance using a Microsoft Surface Tablet computer and Stylus Pen, which more closely duplicates natural line drawing with a pen and paper.

## Supporting Information

S1 Table TBI patient characteristics. (DOCX)
