# Correction: A Computerized Test of Design Fluency

**DOI:** 10.1371/journal.pone.0158933

**Published:** 2016-07-05

**Authors:** The PLOS ONE Staff

In Table 2, the rows "Experiment," "Exp. 1," "Exp. 2/3," and "Exp. 4" are erroneously duplicated within the table. Please see the correct Table 2 here. The publisher apologizes for the error.

**Table 2:** Participant characteristics.

Experiment	Group	N	Ages (yrs)	Education (yrs)	C-use scale	Male (%)
**Exp. 1**	Control/ Normative	180	18–82; 41.6 (21.4)	10–20; 14.6(2.1)	5.09	59%
**Exp. 2/3**	Control/ Malinger	55/52	18–46; 26.2 (5.5)	12–18; 14.8 (1.4)	5.86	51%
**Exp.4**	mTBI	24	20–61; 34.1(11.5)	10–18; 13.5(1.4)	5.04	100%
	sTBI	4	35–52;45.0(7.1)	12–16; 13.0(1.2)	5.25	75%

C-use = computer-use scale; mTBI = mild TBI. sTBI = severe TBI. Range, mean, and variance are shown for age and education.
