# Supplementary material for: A Computerized Test of Design Fluency
Source: PLoS One. 2016 May 3;11(5):e0153952. doi: 10.1371/journal.pone.0153952 (PMC4854436; doi:10.1371/journal.pone.0153952)
Supplement: S1 Table — (DOCX) [file pone.0153952.s001.docx]

| **Table S1. TBI Patient Characteristics** | | | | |
| --- | --- | --- | --- | --- |
|  | Age | C-use | Severity | PCL |
| PA01 | 24 | 5 | Mild | 54 |
| PA02 | 28 | 4 | Mild | 66 |
| PA03 | 57 | 3 | Mild | 56 |
| PA04 | 31 | 4 | Mild | 28 |
| PA05 | 41 | 4 | Mild | 45 |
| PA06 | 20 | 7 | Mild | 41 |
| PA07 | 25 | 8 | Mild | N/A |
| PA08 | 28 | 7 | Mild | 47 |
| PA09 | 25 | 4 | Mild | 57 |
| PA10 | 29 | 6 | Mild | 54 |
| PA11 | 47 | 7 | Mild | 52 |
| PA12 | 28 | 5 | Mild | 43 |
| PA13 | 29 | 5 | Mild | 27 |
| PA14 | 61 | 4 | Mild | 52 |
| PA15 | 27 | 7 | Mild | 72 |
| PA16 | 48 | 4 | Mild | 59 |
| PA17 | 49 | 8 | Mild | 47 |
| PA18 | 28 | 1 | Mild | 68 |
| PA19 | 39 | 4 | Mild | 64 |
| PA20 | 25 | 2 | Mild | 72 |
| PA21 | 45 | 3 | Mild | 60 |
| PA22 | 23 | 8 | Mild | 67 |
| PA23 | 29 | 6 | Mild | 41 |
| PA24 | 28 | 5 | Mild | 46 |
| PA25 | 35 | 5 | Severe | 59 |
| PA26 | 46 | 2 | Severe | 42 |
| PA27 | 52 | 7 | Severe | 27 |
| PA28 | 47 | 7 | Severe | 50 |
| C-use = computer use score; Severity = severity of traumatic brain injury; PCL = Post-traumatic Stress Disorder Check List, total score. | | | | |
